# Alternative processing technology for the preparation of carbonized Zingiberis Rhizoma by stir-frying with sand

**DOI:** 10.1080/13880209.2019.1711431

**Published:** 2020-01-22

**Authors:** Shen Mei-Yu, Wang Jia-Li, Shi Hai-Pei, Yan Hui, Chen Pei-Dong, Yao Wei-Feng, Bao Bei-Hua, Zhang Li

**Affiliations:** a School of Pharmacy, Nanjing University of Chinese Medicine, Nanjing, Jiangsu, People’s Republic of China; b Jiangsu Collaborative Innovation Center of Chinese Medicinal Resources Industrialization, and National and Local Collaborative Engineering Center of Chinese Medicinal Resources Industrialization and Formulae Innovative Medicine, Nanjing University of Chinese Medicine, Nanjing, Jiangsu, People’s Republic of China

**Keywords:** Stir-frying processing technology, fingerprint, gingerols, shogaols

## Abstract

**Context:**

Carbonized ginger, a type of charry herb, has been used as a hemostatic medicine since ancient times. However, there are some serious problems such as inhomogeneous heating and emitting smoke during processing with traditional stir-frying method.

**Objective:**

To investigate the feasibility to obtain carbonized ginger by stir-frying with sand instead of stir-frying method.

**Materials and methods:**

Dried-ginger (100 g) was processed by stir-frying for 30 min at 270 ± 10 °C, or by stir-frying with sand (1:10, w/w) for 8 min at 240 ± 5 °C. The HPLC fingerprint was established for two samples. The adsorption capacity and major components including tannins, gingerols, shogaols and gingerone were quantitated by UV and HPLC, respectively. The hemostatic effect by prothrombin time (PT) and activated partial thromboplastin time (APTT) was evaluated *in vitro*.

**Results:**

The similarity of the two samples for HPLC fingerprints was >0.93. The sand-fried samples showed significantly higher adsorption capacity compared with the stir-fried samples (4.915 vs. 4.593 mg/g; *p <* 0.05) and higher contents of major components (4.698 vs. 3.930 mg/g, 1.352 vs. 1.144 mg/g, 2.419 vs. 2.095 mg/g, 0.666 vs. 0.568 mg/g and 1.083 vs. 0.911 mg/g for tannins, gingerone, 6-shogaol, 8-shogaol and 10-shogaol, respectively; *p <* 0.05); while no significant differences were seen for 6-gingerol, 8-gingerol and 10-gingerol (*p >* 0.05). The PT and APTT values were similar between the stir-fried and sand-fried test groups and significantly lower compared to controls (*p <* 0.05).

**Conclusions:**

The carbonizing process by stir-frying with sand is superior to the stir-frying method for carbonized ginger.

## Introduction

*Zingiber officinale* Roscoe (Zingiberaceae), commonly known as ginger, is one of the most important plant species, and of high value in the world for its various uses such as spice and medicine (Sharifi-Rad et al. 2017; Arablou and Aryaeian 2018; Mao et al. 2019). In China, ginger has been used as a traditional Chinese medicine for thousands of years. The processed ginger products as listed in the 2015 edition of Chinese Pharmacopoeia (Chinese Pharmacopoeia Commission 2015) mainly include fresh ginger, dried ginger, prepared dried ginger and carbonized ginger. Carbonized ginger, also called ‘Jiangtan’, is the carbonized product of the pieces of dried ginger by simple stir-frying technology, and traditionally used for the treatment of hematochezia, metrorrhagia and metrostaxis with known warming meridian and hemostasis effects in accordance with the Traditional Chinese Medicine (TCM) theory. However, there are some concerns about the processing method such as an inhomogeneous heating process and emitting smoke, which are potentially harmful to the quality of carbonized ginger and the health of workers.

The stir-frying with sand (also called scalding with sand) technology used for the preparation of prepared dried ginger is also an important processing method for Chinese medicine, which utilizes hot sand heated with strong fire to process medicinal materials. This method has the advantages of fast and uniform heat transfer, larger contact area, controllable fire intensity and high yield and efficiency, in addition to reducing the generation of smoke during the operation.

The polyphenols are the major chemical components in ginger and consist of gingerols, shogaols and paradols. In fresh ginger, gingerols are the main polyphenols and include 6-gingerol, 8-gingerol and 10-gingerol. After heat treatment, gingerols can be transformed into corresponding shogaols (Li et al. 2016; Ghasemzadeh et al. 2018; Jung et al. 2018; Ko et al. 2019). In addition, tannins are considered as the active hemostatic component of charcoal drugs (Deng et al. 2017; Li et al. 2017).

This study determined the feasibility of preparing carbonized ginger by stir-frying with sand instead of simple stir-frying method as assessed by comparing adsorption capacity, contents of major chemical components, fingerprints and pharmacological effects of carbonized ginger prepared by the two carbonizing methods.

## Materials and methods

### Materials and major reagents

Dried gingers were purchased from Anhui Bozhou Medicinal Materials Market and identified as dried roots of *Zingiber officinale* by Professor Yan Hui, Department of Traditional Chinese Drug Identification, Nanjing University of Chinese Medicine, Nanjing, China. Voucher specimens (Nos. GJ20171010-01 to GJ20171010-10) were preserved in NJUCM herbaria. Methylene blue was purchased from China Pharmaceutical (Group) Shanghai Chemical Reagent Company. Gallic acid reference standard (Batch No. 19630) was purchased from Shanghai Jingchun Reagent Co., Ltd. The reference standards of Zingerone (JBZ180323-032), 6-gingerol (JBZ180110-027), 8-gingerol (JBZ180209-028), 6-shogaol (JBZ180208-030), 8-shogaol (JBZ171129-03) and 10-shogaol (JBZ180108-044) were purchased from Nanjing Jinyibai Bio Technology Co., Ltd. (Nanjing, Jiangsu, China) (all purity ≥98%, by HPLC). 10-Gingerol (P2DN6F6295) was purchased from Shanghai Yuanye Biotechnology Co., Ltd. (Shanghai, China) (purity ≥98%, by HPLC). Acetonitrile and methanol were chromatographically pure (Jiangsu Hanbang Technology Co., Ltd., Huaian, Jiangsu, China). Ultrapure water was obtained with a Milli-Q ultrapure water system (Millipore Inc., USA). Sodium citrate (Batch No. 20170407) was purchased from Tianjin Jinhui Taiya Chemical Reagent Co., Ltd. (Tianjin, China)

### Instrumentation

The following instruments and equipment were used in this study: e2695 high performance liquid chromatography equipped with a 2998 PDA UV detector (Waters, Milford, MA), BP211D electronic balance (Sedolis Scientific Instrument Co., Ltd., Beijing, China), Anke-TGL-16B centrifuge (Shanghai Anting Scientific Instrument Factory, Shanghai, China), UV-1600 spectrophotometer (Shanghai Mapada Instrument Co., Ltd., Shanghai, China), KH-500DB CNC ultrasonic cleaner (Kunshan Hechuang Ultrasonic Instrument Co., Ltd., Kunshan, China), XFB-200 high-speed Chinese medicine grinder (Jishou City Loyalty Pharmaceutical Machinery Factory, Jishou, China), and AYW8000 semiautomatic coagulation analyzer (Taizhou Steellex Biotechnology Co., Ltd., Taizhou, China).

### Preparation of carbonized ginger samples

#### Preparation of stir-fried samples

Dried ginger (100 g) was weighed in duplicate and processed by simple stir-frying for 30 min at 270 ± 10 °C until the black charred surface and brown inner part was observed.

#### Preparation of sand-fried samples

Dried ginger (100 g) was weighed in duplicate and processed by stir-frying with sand (1:10, w/w) for 8 min at sand temperature 240 ± 5 °C.

### Determination of adsorption capacity by methylene blue adsorption test

The methylene blue adsorption test was performed to determine the adsorption capacity of the stir-fried and sand-fried samples according to the national standard of China: Test methods of wooden activated carbon—determination of methylene blue adsorption (GBT12496.10-1999), as follows (SAC 1994).

All samples and methylene blue were dried for 1 h in an oven at 105 ± 1 °C and transferred to a desiccator to cool for 0.5 h. This step was repeated until the sample weights were constant. The water content of each sample was calculated based on the reduced weights.

Methylene blue (0.16 g) (14.1% water) was added to 6.5 L phosphate buffer solution (pH 7), and mixed. Aliquots of this solution at different volumes of 0, 0.5, 1.0, 2.0, 3.0, 4.0, 5.0 and 6.0 mL were pipetted precisely into a 25 mL round-bottomed flask, and diluted to volume with phosphate buffer solution (pH 7) and then mixed by shaking. The absorbance was measured with UV spectrophotometer at 665 nm, and the calibration curve was generated by plotting the absorbance values versus concentration of methylene blue.

The test solution was prepared by adding 0.1 g carbonized ginger powder to 25 mL of 0.0244 g/L methylene blue solution and shaken at 150 rpm for 1 h to achieve adsorption. The absorbance was measured as described above and used to calculate the amount of unabsorbed methylene blue by interpolating from the calibration curve. The adsorption ratio was calculated according to the following Equation (1).

(1) Adsorption capacity (mg/g)=(m–A)/S

where *m* is the initial amount of methylene blue in the test solution (mg); *A* is the amount of unabsorbed methylene blue (mg); *S* is the amount of sample (g).

### Determination of tannins

The gallic acid standard was used to determine tannins in the carbonized ginger samples according to the Chinese Pharmacopoeia (2015) as follows. Aliquots of gallic acid solution (0.0502 mg/mL) at different volumes of 0.5, 1.0, 1.5, 2.0, 2.5 and 3.0 mL were added to a 25 mL round-bottomed flask containing 1 mL of phosphomolybdiumtungstic acid (PMTA) solution, diluted to 13 mL with distilled water and then diluted to volume with 29% sodium carbonate solution, and finally analyzed 30 min later by UV spectrophotometer at 760 nm. The calibration curve was calculated.

Carbonized ginger powder (3.0 g) was weighed out into a 250 mL round-bottomed flask and 150 mL of distilled water was added. After ultrasonic oscillation (200 W) for 10 min, the resulting solution was cooled under running water, diluted to volume with distilled water, and allowed to settle prior to filtration. The first 50 mL of filtrate was discarded and the subsequent 20 mL filtrate was collected into a 100 mL round-bottomed flask, diluted to volume with distilled water. The solution was the test solution.

The test solution (2.0 mL) and 1 mL of PMTA solution was added to a 25 mL round-bottomed flask, diluted to 13 mL with distilled water and then diluted to volume with 29% sodium carbonate solution, and finally analyzed 30 min later as described above. The total polyphenols were calculated from the above calibration curve.

Casein (0.6 g) was added to 25.0 mL of test solution and oscillated for 1 h (30 °C), then filtered. The first 10 mL of filtrate was discarded, and 2.0 mL of the subsequent filtrate was collected and mixed with 1 mL of PMTA solution and 10 mL of distilled water and diluted to volume with 29% sodium carbonate solution. The absorbance was measured 30 min later at 760 nm. The quantity of polyphenols not adsorbed by casein was calculated according to the calibration curve. The percentage content of tannins was calculated according to the following equation:

(2) Content of tannins (mg/g)=(A1–A2)/S

where *A*1 is the content of the total polyphenols (mg); *A*2 is the content of the polyphenols not adsorbed by hide powder (mg); *S* is the amount of sample (g).

### Establishment of HPLC fingerprint of carbonized ginger

#### Chromatographic conditions

HPLC fingerprint of carbonized ginger was performed using the Waters e2695 equipped with a 2998 PDA UV detector. All samples were analyzed by a RP column (C18 analytical column, 4.6 mm × 250 mm, 5 μm, Waters, Milford, MA) using gradient elution with acetonitrile (A) and water (B) (0–30 min, 35–70% A; 30–50 min, 70–90% A; 50–60 min, 90% A). The flow rate was 1 mL/min and the column temperature was set at 30 °C. The sample injection volume was 10 μL. The UV spectra were acquired from 200 to 400 nm with UV detection at 240 nm.

#### Preparation of standard solution

The reference standard solutions of 10-shogaol, 8-shogaol, 10-gingerol, 6-shogaol, 8-gingerol, 6-gingerol and zingerone were prepared in 75% methanol at a concentration of 4.780, 7.475, 1.618, 2.080, 0.799, 6.770 and 1.912 mg/mL, respectively.

#### Preparation of test solution

A sufficient amount of carbonized ginger was ground into coarse powder and passed through a #3 sieve. Approximately 0.5 g sample of carbonized ginger powder was accurately weighed into a stopper conical flask, and 20 mL of 75% methanol was added and weighed. The solution was ultrasonicated with a power of 100 W at a frequency of 40 kHz for 40 min, cooled and weighed again, replenished the loss of solvent with 75% methanol and mixed well for filtration (0.45 μm). The filtrate was used as the test solution (Shi et al. 2019).

#### Validation of the HPLC fingerprint method

The precision was determined by injecting the same sample solution for six times within 1 day. The reproducibility was determined by analyzing six separate sample solutions extracted from the same carbonized ginger batch. The stability test was evaluated by injecting the same sample solution at 0, 2, 4, 6, 8 and 10 h after preparation.

#### Similarity analysis

The Similarity Evaluation System for Chromatographic Fingerprint of Traditional Chinese Medicine (Version 2012, Chinese Pharmacopoeia Commission), recommended by China Food and Drug Administration (CFDA), was used for the evaluation of similarity across chromatograms of different samples, and the correlation coefficients were included in the analysis.

### Determination of active ingredients in carbonized ginger

#### Chromatographic conditions

The HPLC conditions were the same as described above for establishment of HPLC fingerprint except for UV detection at 280 nm for gingerone, 6-gingerol and 8-gingerol and at 220 nm for 6-shogaol, 10-gingerol, 8-shogaol and 10-shogaol.

#### Preparation of standard solution

A total of seven standard stock solutions were prepared in 75% methanol at a concentration of 4.780 mg/mL (10-shogaol), 7.475 mg/mL (8-shogaol), 1.618 mg/mL (10-gingerol), 2.080 mg/mL (6-shogaol), 0.799 mg/mL (8-gingerol), 6.770 mg/mL (6-gingerol) and 1.912 mg/mL (zingerone), respectively. The working standard solutions were prepared daily by mixing and diluting the respective stock solutions with 75% methanol.

#### Preparation of test solution

The test solution was prepared as described above for establishment of HPLC fingerprint.

#### Validation of the HPLC method

The linearity of the HPLC method was evaluated against the calibration curve. The precision was determined by injecting the same sample solution for six times within 1 day. The stability was evaluated by injecting the same sample solution at 0, 2, 4, 6, 8 and 10 h after preparation. The reproducibility was determined by analyzing six separate sample solutions extracted from the same carbonized ginger batch. Meanwhile, the recovery experiments were performed according to Chinese Pharmacopoeia (2015) to investigate the accuracy of the developed method.

### *In vitro* blood coagulation test

#### Preparation of test solution

Approximately 30 g of carbonized ginger were weighed and decocted twice with water for 30 min (300 mL water) and 20 min (200 mL water), respectively. The two decoctions were combined, filtered, and finally concentrated to get a 30 mL water extract.

#### Determination of activated partial thromboplastin time (APTT) and prothrombin time (PT)

One healthy New Zealand White rabbit supplied by Nanjing Qinglong Experimental Animal Center (certificate number 20170702) was used. Animal welfare and experimental procedures strictly adhered to the Guide for the Care and Use of Laboratory Animal and were approved by the Animal Ethics Committee of Nanjing University of Chinese Medicine prior to the experiment. After the rabbit was anesthetized with 6% pentobarbital sodium, the required blood samples were withdrawn from the central ear artery into the appropriate tubes containing 3.8% sodium citrate (anticoagulant to blood ratio 1: 9, v/v). All blood samples were centrifuged at 3000 rpm for 10 min at 15 °C to obtain plasma. APTT and PT were measured using the commercial PT (Lot. 20170815) and APTT (Lot. 20170815) kits supplied by Beijing Steellex Scientific Instrument Company, respectively.

### Statistical analysis

Statistical comparisons between groups were performed by Student’s *t*-test, one-way analysis of variance (ANOVA) using PRISM 6.0 (Trial, GraphPad Software Inc., La Jolla, CA). *p <* 0.05 was considered statistically significant. Data are presented as mean ± standard deviation (SD).

## Results

### Adsorption capacity

The mean absorption of methylene blue was significantly higher in the sand fried samples than in the stir-fried samples (4.915 vs. 4.593 mg/g, *p <* 0.05), indicating a greater adsorption capacity for the sand fried samples (Figure S1 and Table S1).

### Content of tanninoids

The mean tannins content was significantly higher in the sand fried samples than in the stir-fried samples (4.698 vs. 3.930 mg/g, *p <* 0.05; Figure S2 and Table S2).

### HPLC fingerprints

#### Establishment of the HPLC fingerprint

**Figure 1.**
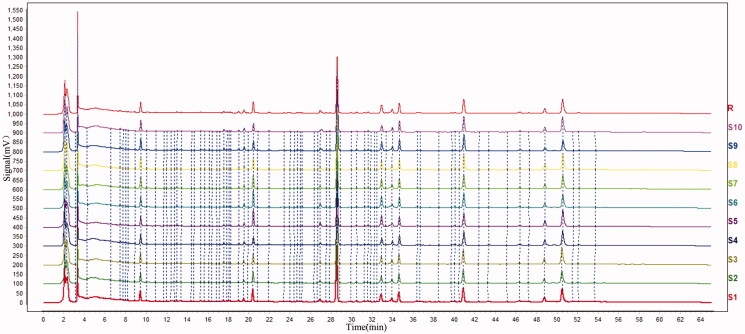
Overlaid HPLC chromatograms of stir-frying with sand method-processed samples.

**Figure 2.**
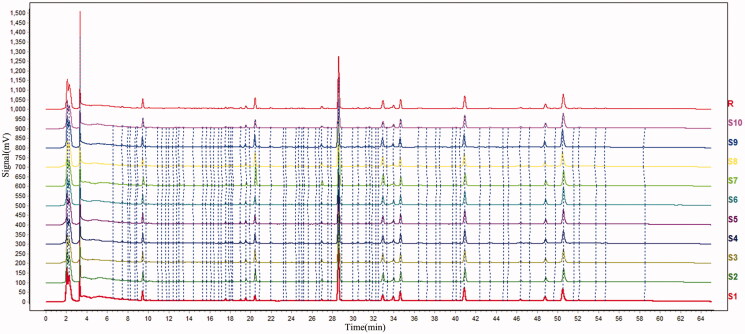
Overlaid HPLC chromatograms of stir-frying method-processed samples.

**Figure 3.**
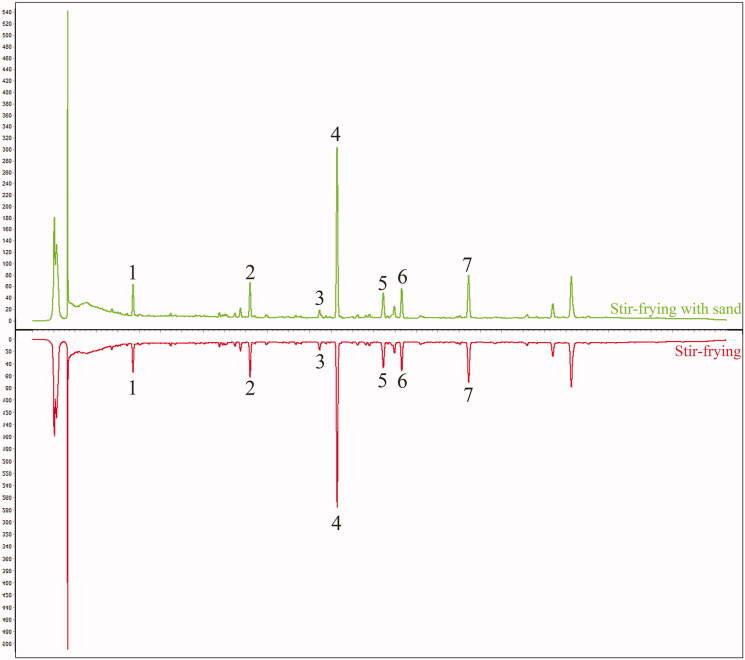
The reference chromatogram of two kind samples.

HPLC chromatographic conditions were optimized based on references and actual samples. A total of 20 batches of carbonized ginger samples (each 10 batches for the stir-fried and sand fried samples) were analyzed by HPLC. As show in Figures 1–3, HPLC chromatograms of the stir-fried and sand fried samples are very comparable. There are 11 common peaks in the HPLC fingerprints with good resolution. Seven of these 11 peaks were identified by comparing UV profile and retention time with those of the respective reference standards as zingerone, 6-gingerol, 8-gingerol, 6-shogaol, 10-gingerol, 8-shogaol and 10-shogaol (from Nos. 1 to 7), respectively. Peak 4 (6-shogaol) was selected from these identified peaks as the reference peak (S) due to its high content, high intensity and moderate retention time in chromatograms. The relative retention time (RRT) and relative peak area (RPA) of remaining six peaks with respect to the reference peak are presented in Table 1.

**Table 1.** RRT and RPA of common peaks of two kinds samples.

Peak no.	Stir-frying		Stir-frying with sand	
	RRT	RPA	RRT	RPA
1	0.331 ± 0.001	0.160 ± 0.012	0.331 ± 0.000	0.169 ± 0.015
2	0.683 ± 0.000	0.058 ± 0.002	0.683 ± 0.000	0.054 ± 0.002
3	0.715 ± 0.000	0.203 ± 0.076	0.715 ± 0.000	0.195 ± 0.057
4	0.942 ± 0.000	0.060 ± 0.017	0.942 ± 0.000	0.059 ± 0.016
5	1.000 ± 0.000	1.000 ± 0.000	1.000 ± 0.000	1.000 ± 0.000
6	1.151 ± 0.000	0.206 ± 0.039	1.151 ± 0.000	0.192 ± 0.035
7	1.188 ± 0.000	0.101 ± 0.022	1.186 ± 0.004	0.104 ± 0.016
8	1.212 ± 0.000	0.193 ± 0.005	1.213 ± 0.003	0.194 ± 0.003
9	1.431 ± 0.001	0.309 ± 0.010	1.431 ± 0.000	0.319 ± 0.008
10	1.707 ± 0.001	0.120 ± 0.012	1.707 ± 0.001	0.113 ± 0.021
11	1.768 ± 0.001	0.421 ± 0.061	1.768 ± 0.001	0.383 ± 0.060

The relative retention time (RRT) was obtained by dividing the retention time of the component by the retention time of the reference peak (Peak 4). And the relative peak area (RPA) was obtained by dividing the peak area of the component by the peak area of the reference peak (Peak 4). Data were expressed as the mean ± SD (*n* = 10).

For the method validation, the RSD values calculated from RRT and RPA were less than 4%, demonstrating good instrumental and sampling precision, and adequate extraction method and chromatographic conditions (Tables S3–S5).

#### Similarity analysis

As shown in Table 1, there were no significant differences in RRT and RPA between the stir-fried and sand fried samples (*p >* 0.05).

Based on the similarity analysis of chromatographic profiles across 20 batches of carbonized ginger samples, the similarity coefficient between batches was more than 0.93, indicating a highly significant similarity across all samples. When compared to the chromatograms of 10 batches of stir-fried samples, the overall similarity coefficient for the sand fried samples ranged from 0.938 to 0.987 (Table 2), indicating no difference in HPLC fingerprints between the sand fried samples and stir-fried samples.

**Table 2.** Similarities of two kinds samples.

Stir-frying	Similarity	Stir-frying with sand	Similarity
1	0.944	1	0.989
2	0.932	2	0.973
3	0.991	3	0.930
4	0.981	4	0.966
5	0.992	5	0.970
6	0.964	6	0.979
7	0.975	7	0.957
8	0.986	8	0.977
9	0.986	9	0.974
10	0.990	10	0.968

The similarities were obtained by the Similarity Evaluation System for Chromatographic Fingerprint of TCM (Version 2012), recommended by CFDA. The similarity coefficient greater than 0.9 indicates that there were no differences in HPLC fingerprints between the two samples.

### Quantitative measurements of 7 compounds

#### Method validation

Linear regression analysis of the peak area (*y*-axis) for each compound (10-shogaol, 8-shogaol, 10-gingerol, 6-shogaol, 8-gingerol, 6-gingerol and zingerone) against the concentration (*x*-axis, mg/mL) of the respective standard solution indicated a good linearity in the analyzed concentration ranges (Table S6). The precision (% RSD) for all analytes ranged from 0.14 to 0.20%. The stability (% RSD) was ≤1.55%. The reproducibility (% RSD) ranged from 1.52 to 3.63%. The recovery values were in the range of 86.32 to 96.78% with %RSD <4.73% (Table S7). The validation results met acceptance criteria as per Chinese Pharmacopoeia (2015).

#### Quantitative results of seven components

The quantitative results of seven components in the stir-fried and sand fried samples are shown in Table 3. The mean gingerone, 6-shogaol, 8-shogaol and 10-shogaol levels in the sand fried samples were significantly higher than those in the stir-fried samples (*p <* 0.05). However, no significant differences between sand fried and stir-fried samples were seen for 6-gingerol, 8-gingerol and 10-gingerol levels (*p >* 0.05).

**Table 3.** The contents (mg/g) of 7 constituents in 10 batches of two samples (*n* = 10).

Analyte*s*	Stir-frying	Stir-frying with sand
Gingerone	1.144 ± 0.089	1.352 ± 0.260*
6-Gingerol	2.214 ± 0.860	2.394 ± 0.680
8-Gingerol	0.058 ± 0.018	0.073 ± 0.023
6-Shogaol	2.095 ± 0.079	2.419 ± 0.430*
10-Gingerol	1.431 ± 0.350	1.545 ± 0.380
8-Shogaol	0.568 ± 0.021	0.666 ± 0.130*
10-Shogaol	0.911 ± 0.036	1.083 ± 0.210*

Data are expressed as the mean ± SD (*n* = 10). *p < 0.05 compared with the stir-frying group.

### Effects on coagulation function in vitro

**Figure 4.**
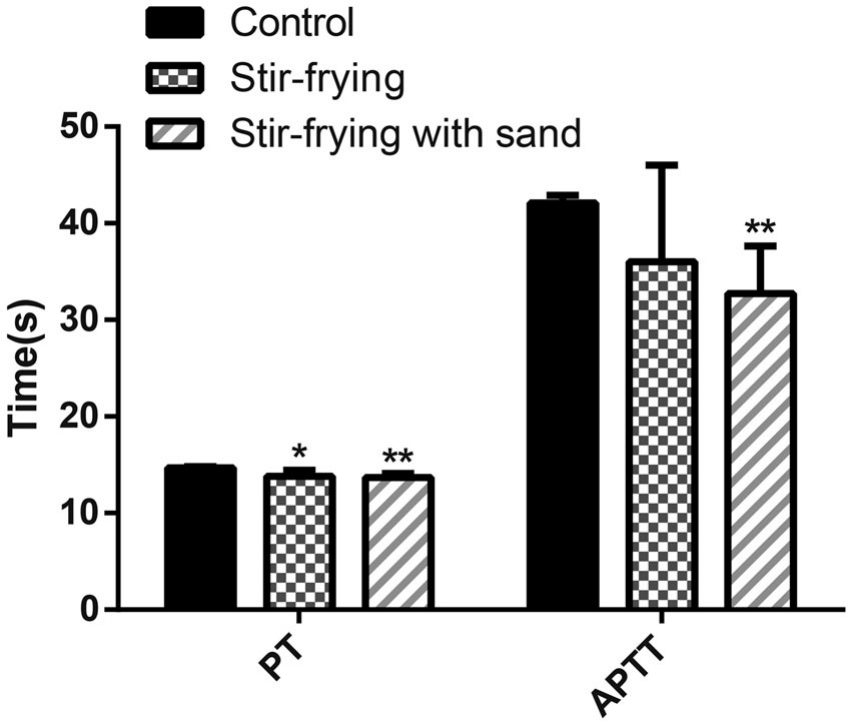
The hemostatic effects of two samples. The levels of PT and APTT were measured by AYW8000 semi-automatic coagulation analyzer (*n* = 9). Data were expressed as the mean ± SD (*n* = 9). *p < 0.05 and **p < 0.01, compared with the control group.

The PT and APTT values were 13.96 s and 38.23 s in the stir-fried test group and 13.75 s and 34.15 s in the sand fried test group, which were significantly lower than the 14.70 s and 41.70 s observed in the blank group (all *p <* 0.05), but no differences between the two test groups were seen (*p >* 0.05), indicating no significant effect of different carbonization methods on coagulation function. These results are given in Figure 4.

## Discussion

Since CDER Guidance for Industry: Botanical Drug Products in 2004 (Center for Drug Evaluation and Research 2004) was published, the chromatographic fingerprint analysis technique has been recognized as an effective and powerful tool for the quality control of botanical drug products. This technique has been introduced into the three ginger-based traditional Chinese herbal drugs (Deng et al. 2015). In our experiment, an improved HPLC fingerprint was established to compare carbonized ginger samples from two different processing methods. The extracting condition of carbonized ginger was optimized. 75% Methanol was finally selected as the extraction solvent and ultrasonic treatment was conducted for 40 min to prepare the test solution. Although the effect of methanol/water mobile phase system was comparable with acetonitrile/water mobile phase system, a longer time was taken with methanol/water mobile phase system; therefore, acetonitrile/water system was selected for experiment. Based on the full wavelength scanning, the detection at 240 nm provided better results with respect to the number and intensity of peaks. The main characteristic peaks were consistent and the similarity assessment showed no significant differences between the stir-fried and sand-fried samples.

In accordance with Chinese Pharmacopoeia (2015), both 6-gingerol and gingerone have been identified as the markers for quality control of carbonized ginger; the HPLC and TLC methods are used for the assay of 6-gingerol (the pharmacopoeial limit of not less than 0.050%) and the identification of gingerone, respectively. Based on the results of our fingerprint analysis and the published literatures (Park and Jung 2016; Koch et al. 2017; You et al. 2019), the HPLC method for the quantitation of 7 main components in carbonized ginger was established. To ensure the accuracy and sensitivity of the method, the maximum absorption wavelength of each component (280 nm for gingerone, 6-gingerol and 8-gingerol; 220 nm for 6-shogaol, 10-gingerol, 8-shogaol and 10-shogaol) was selected as the detection wavelength. For the sand fried samples, the observed significant increases in gingerone, 6-shogaol, 8-shogaol and 10-shogaol levels as measured by the established HPLC method may be explained by the fact that the active hydrogen of C4 is easily dehydrated together with the hydroxyl group of C5 to form shogaol under heated condition, and the latter can be further subjected to retro Diels–Alder reaction to form gingerone (Semwal et al. 2015; Ghasemzadeh et al. 2018; Jung et al. 2018). In addition, tannins are considered as the active hemostatic component of charcoal drugs (Deng et al. 2017; Li et al. 2017); in our study, the adsorption capacity and tannins level were also in favor of the sand-fried samples versus the stir-fried samples. These differences may reflect fast and uniform heat transfer, as well as larger contact area with carbonizing by stir-frying with sand.

In addition, we also compared the two carbonizing process in terms of the pharmacological effects of carbonized ginger as the carbonizing process by stir-frying is associated with the hemostatic effect of traditional Chinese medicines according to the TCM theory (Oakley and Larjava 2013; Chen et al. 2019). In our study, stir-fried and sand fried carbonized gingers were shown to have hemostatic effect *in vitro*, as indicated by significantly decreased PT and APTT values in the test groups compared with the control group, and no significant differences between the two test groups. Although the sand-fried samples showed significantly higher contents of major components for tannins, gingerone, 6-shogaol, 8-shogaol and 10-shogaol; while no significant differences were seen for 6-gingerol, 8-gingerol and 10-gingerol. As total contents amount to no more than 1%, these components may do not fully reflect pharmacological difference. Then, there is not much pharmacological difference between two samples although these contents are not the same. However, considering the complex coagulation mechanism and the fact that the active components of carbonized ginger have not been fully analyzed, and the action targets of each component have not been illuminated, this study should be considered as preliminary. Further studies are warranted to comprehensively understand the underlying mechanisms of these effects.

## Conclusions

Under the conditions of this study, the carbonizing process by stir-frying with sand may deliver equivalent or better carbonized ginger than the simple stir-frying method, supporting the use of stir-frying with sand for the preparation of carbonized ginger.

## Supplementary Material

Supplementary_Material.docx
