# Supplementary material for: Alternative processing technology for the preparation of carbonized Zingiberis Rhizoma by stir-frying with sand
Source: Pharm Biol. 2020 Jan 22;58(1):131–7. doi: 10.1080/13880209.2019.1711431 (PMC7006685; doi:10.1080/13880209.2019.1711431)
Supplement: Supplementary_Material.docx [file IPHB_A_1711431_SM7430.docx]

**SUPPLEMENTARY MATERIAL**

**Alternative processing technology for the preparation of carbonized Zingiberis Rhizoma by stir-frying with sand**

Shen Mei-yu^a,#^, Wang Jia-Li^a,#^, Shi Hai-pei^a^, Yan Hui^a,b^, Chen Pei-dong^a,b^, Yao Wei-Feng^a,b^, Bao Bei-hua^a,b,^^[[1]](#footnote-1)^*, Zhang Li^a,b,*^

*^a^ School of Pharmacy, Nanjing University of Chinese Medicine, Nanjing, 210023, PR China*

*^b^Jiangsu Collaborative Innovation Center of Chinese Medicinal Resources Industrialization, and National and Local Collaborative Engineering Center of Chinese Medicinal Resources Industrialization and Formulae Innovative Medicine, Nanjing University of Chinese Medicine, Nanjing, 210023, PR China*

* Corresponding author. School of Pharmacy, Nanjing University of Chinese Medicine. E-mail address: baobh@njucm.edu.cn & Zhangli@njucm.edu.cn

# Shen Mei-yu and Wang Jia-Li contributed equally to this work.


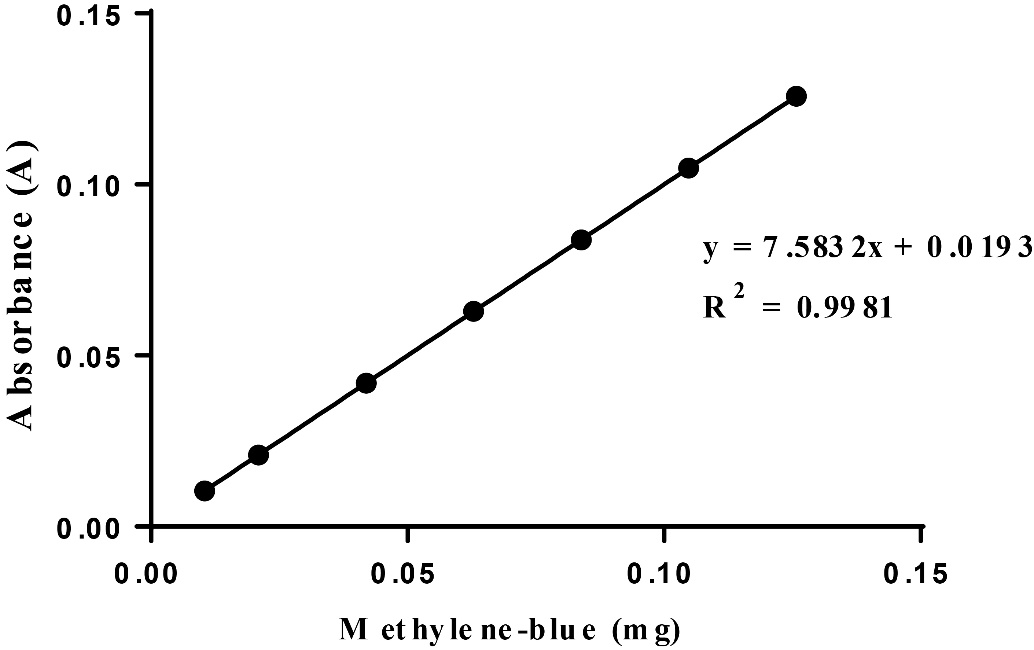


Figure S1. The calibration curve of methyle-blue

Table S1. The adsorption capacity of carbonized ginger (mg/g)

| Sample | Stir-frying | Stir-frying with sand |
| --- | --- | --- |
| 1 | 4.570 | 5.196 |
| 2 | 4.570 | 5.148 |
| 3 | 4.519 | 5.072 |
| 4 | 4.694 | 5.332 |
| 5 | 4.616 | 4.311 |
| 6 | 4.571 | 4.350 |
| 7 | 4.612 | 4.755 |
| 8 | 4.487 | 4.824 |
| 9 | 4.477 | 4.905 |
| 10 | 4.809 | 5.161 |


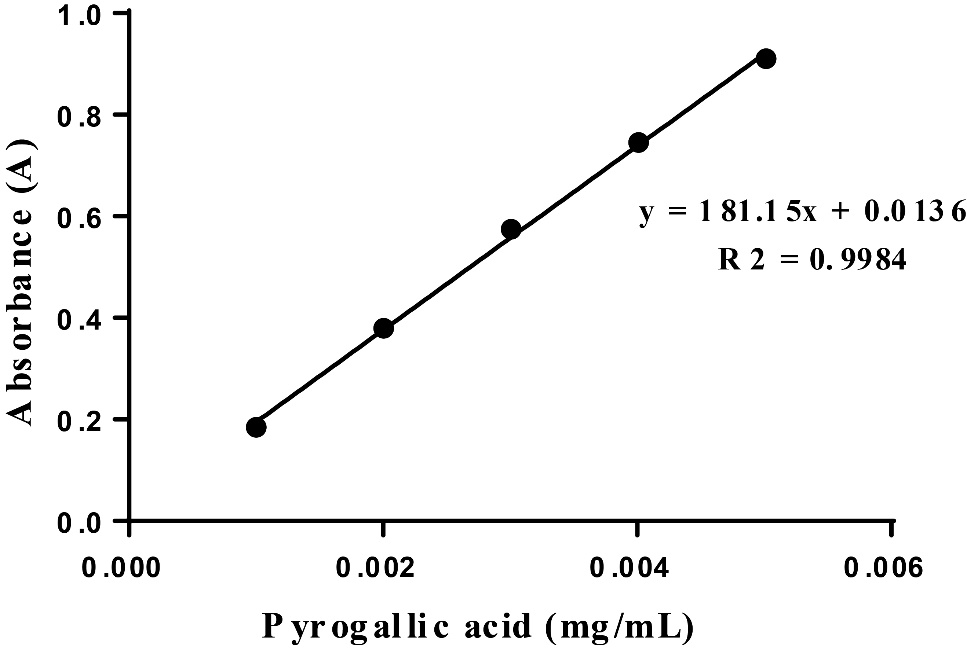


Figure S2. The calibration curve of pyrogallic acid

Table S2. The tannins content of carbonized ginger (mg/g)

| Sample | Stir-frying | Stir-frying with sand |
| --- | --- | --- |
| 1 | 3.440 | 5.197 |
| 2 | 4.037 | 6.022 |
| 3 | 3.865 | 4.903 |
| 4 | 4.809 | 4.770 |
| 5 | 4.360 | 3.864 |
| 6 | 3.349 | 4.004 |
| 7 | 3.307 | 3.730 |
| 8 | 4.075 | 4.944 |
| 9 | 3.700 | 5.143 |
| 10 | 4.357 | 4.399 |

Table S3. Precision of the HPLC fingerprint

| Peak no. | RRT | RSD(%) | RPA | RSD(%) |
| --- | --- | --- | --- | --- |
| 1 | 0.3310±0.0002 | 0.0519 | 0.1669±0.0006 | 0.3515 |
| 2 | 0.6830±0.0001 | 0.0143 | 0.0582±0.0009 | 1.5392 |
| 3 | 0.7146±0.0001 | 0.0162 | 0.1340±0.0003 | 0.2234 |
| 4 | 0.9421±0.0001 | 0.0126 | 0.0416±0.0003 | 0.7011 |
| 5 | 1.0000±0.0000 | 0.0000 | 1.0000±0.0000 | 0.0000 |
| 6 | 1.1515±0.0001 | 0.0061 | 0.1661±0.0010 | 0.6237 |
| 7 | 1.1876±0.0001 | 0.0063 | 0.1182±0.0006 | 0.5365 |
| 8 | 1.2119±0.0001 | 0.0077 | 0.1954±0.0002 | 0.0818 |
| 9 | 1.4313±0.0002 | 0.0127 | 0.3225±0.0006 | 0.1893 |
| 10 | 1.7073±0.0002 | 0.0107 | 0.1409±0.0008 | 0.5574 |
| 11 | 1.7681±0.0002 | 0.0131 | 0.4867±0.0032 | 0.6572 |

Table S4. Stability of the HPLC fingerprint

| Peak no. | RRT | RSD(%) | RPA | RSD(%) |
| --- | --- | --- | --- | --- |
| 1 | 0.3309±0.0002 | 0.0461 | 0.1667±0.0017 | 1.0280 |
| 2 | 0.6830±0.0001 | 0.0180 | 0.0579±0.0003 | 0.5936 |
| 3 | 0.7147±0.0002 | 0.0224 | 0.1331±0.0018 | 1.3308 |
| 4 | 0.9421±0.0001 | 0.0146 | 0.0415±0.0004 | 1.0836 |
| 5 | 1.0000±0.0000 | 0.0000 | 1.0000±0.0000 | 0.0000 |
| 6 | 1.1515±0.0001 | 0.0053 | 0.1666±0.0005 | 0.2759 |
| 7 | 1.1815±0.0147 | 1.2482 | 0.1183±0.0004 | 0.3796 |
| 8 | 1.2118±0.0002 | 0.0167 | 0.1953±0.0004 | 0.1878 |
| 9 | 1.4310±0.0005 | 0.0353 | 0.3217±0.0013 | 0.3984 |
| 10 | 1.7070±0.0006 | 0.0370 | 0.1391±0.0024 | 1.7051 |
| 11 | 1.7677±0.0008 | 0.0467 | 0.4795±0.0094 | 1.9666 |

Table S5. Reproducibility of the HPLC fingerprint

| Peak no. | RRT | RSD(%) | RPA | RSD(%) |
| --- | --- | --- | --- | --- |
| 1 | 0.3310±0.0001 | 0.0362 | 0.1642±0.0015 | 0.8949 |
| 2 | 0.6831±0.0002 | 0.0271 | 0.0547±0.0010 | 1.8772 |
| 3 | 0.7147±0.0003 | 0.0366 | 0.1351±0.0040 | 2.9877 |
| 4 | 0.9421±0.0001 | 0.0147 | 0.0433±0.0009 | 2.1557 |
| 5 | 1.0000±0.0000 | 0.0000 | 1.0000±0.0000 | 0.0000 |
| 6 | 1.1514±0.0002 | 0.0163 | 0.1653±0.0011 | 0.6946 |
| 7 | 1.1874±0.0003 | 0.0246 | 0.1173±0.0008 | 0.7230 |
| 8 | 1.2117±0.0004 | 0.0310 | 0.1952±0.0003 | 0.1523 |
| 9 | 1.4309±0.0009 | 0.0649 | 0.3218±0.0025 | 0.7734 |
| 10 | 1.7067±0.0014 | 0.0839 | 0.1294±0.0025 | 1.9220 |
| 11 | 1.7675±0.0016 | 0.0930 | 0.4577±0.0127 | 2.7716 |

Table S6. Calibration curves of seven analytes

| Reference substance | Standard curve | r | Linear range (μg/ml) |
| --- | --- | --- | --- |
| Gingerone | y=17557925.2336x+14184.8958 | 0.9998 | 3.585～57.36 |
| 6-gingerol | y=10645278.7175x+14948.7292 | 0.9998 | 6.347～101.6 |
| 8-gingerol | y=113842635.9562x+4526.1875 | 0.9998 | 0.1997～3.196 |
| 6-shogaol | y=86494693.0142x+167457.1458 | 0.9998 | 7.800～124.8 |
| 10-gingerol | y=22484045.7488x+44002.8333 | 0.9998 | 8.090～129.4 |
| 8-shogaol | y=62117422.5914x+50086.625 | 0.9998 | 3.738～59.80 |
| 10-shogaol | y=65725431.4745x+55621.1042 | 0.9998 | 3.884～62.14 |

Table S7. Precision, Stability, Reproducibility and Recovery of seven analytes

| Analytes | Precision | Stability | Reproducibility | | Recovery | |
| --- | --- | --- | --- | --- | --- | --- |
|  | RSD (%) | RSD (%) | Content (%) | RSD (%) | Mean | RSD (%) |
| Gingerone | 0.17 | 0.92 | 0.1091 | 2.45 | 96.78 | 1.59 |
| 6-gingerol | 0.14 | 0.62 | 0.1323 | 1.52 | 90.95 | 3.04 |
| 8-gingerol | 0.19 | 0.74 | 0.0039 | 3.63 | 96.91 | 3.56 |
| 6-shogaol | 0.15 | 0.87 | 0.1909 | 1.69 | 92.54 | 2.51 |
| 10-gingerol | 0.20 | 1.55 | 0.1012 | 2.00 | 93.21 | 3.59 |
| 8-shogaol | 0.17 | 0.40 | 0.0537 | 1.75 | 87.63 | 1.47 |
| 10-shogaol | 0.14 | 0.21 | 0.0863 | 1.98 | 86.32 | 4.73 |

1. [↑](#footnote-ref-1)
